# p-Cresol Sulfate Caused Behavior Disorders and Neurodegeneration in Mice with Unilateral Nephrectomy Involving Oxidative Stress and Neuroinflammation

**DOI:** 10.3390/ijms21186687

**Published:** 2020-09-12

**Authors:** Chiao-Yin Sun, Jian-Ri Li, Ya-Yu Wang, Shih-Yi Lin, Yen-Chuan Ou, Cheng-Jui Lin, Jiaan-Der Wang, Su-Lan Liao, Chun-Jung Chen

**Affiliations:** 1 Department of Nephrology, Chang Gung Memorial Hospital, Keelung 20401, Taiwan; fish3970@gmail.com; 2 Community Medicine Research Center, Chang Gung Memorial Hospital, Keelung 20401, Taiwan; 3 Kidney Research Center, Chang Gung Memorial Hospital, Taoyuan 33305, Taiwan; 4 School of Medicine, Chang Gung University, Taoyuan 33305, Taiwan; 5 Division of Urology, Taichung Veterans General Hospital, Taichung 40705, Taiwan; fisherfishli@yahoo.com.tw; 6 Department of Family Medicine, Taichung Veterans General Hospital, Taichung 40705, Taiwan; yywang@vghtc.gov.tw; 7 Institute of Clinical Medicine, National Yang Ming University, Taipei 112304, Taiwan; sylin@vghtc.gov.tw; 8 Center for Geriatrics and Gerontology, Taichung Veterans General Hospital, Taichung 40705, Taiwan; 9 Department of Urology, Tungs’ Taichung MetroHarbor Hospital, Taichung 43304, Taiwan; ycou228@gmail.com; 10 Division of Nephrology, Department of Internal Medicine, Mackay Memorial Hospital, Taipei 10449, Taiwan; lincj@mmh.org.tw; 11 Mackay Junior College of Medicine, Nursing and Mangement, Taipei 11260, Taiwan; 12 Children’s Medical Center, Taichung Veterans General Hospital, Taichung 40705, Taiwan; wangjiaander@gmail.com; 13 Department of Industrial Engineering and Enterprise Information, Tunghai University, Taichung 407224, Taiwan; 14 Department of Medical Research, Taichung Veterans General Hospital, Taichung 40705, Taiwan; slliao@vghtc.gov.tw; 15 Department of Medical Laboratory Science and Biotechnology, China Medical University, Taichung 40402, Taiwan; 16 Program in Translational Medicine, College of Life Sciences, National Chung Hsing University, Taichung 40227, Taiwan

**Keywords:** chronic kidney disease, depression, neurodegeneration, neuroinflammation, uremic toxin

## Abstract

Protein-bound uremic toxins, such as p-cresol sulfate (PCS), can be accumulated with declined renal function and aging and is closely linked with central nervous system (CNS) diseases. In the periphery, PCS has effects on oxidative stress and inflammation. Since oxidative stress and inflammation have substantial roles in the pathogenesis of neurological disorders, the CNS effects of PCS were investigated in unilateral nephrectomized C57/BL/6 mice. Unlike intact mice, unilateral nephrectomized mice showed increased circulating levels of PCS after exogenous administration. Upon PCS exposure, the unilateral nephrectomized mice developed depression-like, anxiety-like, and cognitive impairment behaviors with brain PCS accumulation in comparison with the nephrectomy-only group. In the prefrontal cortical tissues, neuronal cell survival and neurogenesis were impaired along with increased apoptosis, oxidative stress, and neuroinflammation. Circulating brain-derived neurotrophic factors (BDNF) and serotonin were decreased in association with increased corticosterone and repressor element-1 silencing transcription factor (REST), regulators involved in neurological disorders. On the contrary, these PCS-induced changes were alleviated by uremic toxin absorbent AST-120. Taken together, PCS administration in mice with nephrectomy contributed to neurological disorders with increased oxidative stress and neuroinflammation, which were alleviated by PCS chelation. It is suggested that PCS may be a therapeutic target for chronic kidney disease-associated CNS diseases.

## 1. Introduction

The incidence and prevalence of chronic kidney disease (CKD) are increasing rapidly worldwide [1]. With disease progression, CKD patients present several comorbidities with high morbidity and mortality rates. Patients with CKD who are undergoing maintenance hemodialysis still have a higher prevalence of neurological complications than those in the general population. Apart from immune and cardiovascular diseases, CKD is closely associated with various central nervous system (CNS) complications, such as Parkinson’s disease, Alzheimer’s disease, cognitive dysfunction, and depression [2,3,4]. Currently, the underlying causes of disease association and the most vulnerable neurological complications are incompletely understood.

CNS diseases are accompanied by a series of biochemical and molecular changes, resulting in acute and long-term impacts on both behavioral and neurological functions. Changes in blood–brain barrier permeability, oxidative stress, neuroinflammation, neurochemicals, neurotrophins, neurogenesis, apoptosis, and synaptic connection are frequently presented in most neurological diseases [5,6]. In rodent models with CKD caused by adenine feeding or a 5/6 nephrectomy, the experimental animals have shown depression- and anxiety-like behaviors concurrent with an impaired blood–brain barrier and the cerebral activation of oxidative stress and neuroinflammation. Those studies suggest that depression and/or anxiety may be the most vulnerable neurological complications of CKD [7,8,9]. CKD and CNS diseases share many commonalities, particularly oxidative stress and inflammation [2,3,4,5,6,7,8,9]. The integration of CKD alterations is believed to promote both the transition and pathogenesis for the development of neurological diseases. It is also likely to acquire pathogenic molecules from CKD, and then transition into neuroactive or neurotoxic surrogates.

CKD or the impairment of renal function tends to cause the retention of various solutes that are normally excreted by the kidneys, and that response negatively interacts with a body’s biological functions. The presence of CNS diseases in CKD patients undergoing maintenance hemodialysis implicates the potential pathogenic contribution of non-dialyzable solutes [2,3,4]. p-Cresol sulfate (PCS), one type of protein-bound uremic toxin, is generated by the liver and intestinal bacteria through the metabolic breakdown of tyrosine and phenylalanine. The gut-microbiota metabolite PCS progressively accumulates during the blood circulation of CKD patients due to its high albumin-binding capacity and has been identified as a potential contributor to clinical complications [10,11,12,13]. In a 5/6 nephrectomy CKD rodent model, the content of PCS is elevated in systemic circulation and the brain tissues, while the increments are decreased by uremic toxin absorbent AST-120 [9,11]. Clinically, a higher cerebrospinal fluid to plasma ratio of PCS is observed in patients diagnosed with Parkinson’s disease [14]. These findings suggest that the association between CKD and CNS diseases may be related to the accumulation, and even brain deposition, of PCS.

PCS displays diverse biological activities. Accumulating evidence has shown that PCS causes cell death and dysfunction centered around oxidative stress, inflammation, impairment of mitochondrial dynamics, and vascular disruption [15,16,17,18,19,20,21]. Thus, both the causative and pathogenic effects of PCS in CNS diseases are highly proposed. The accumulation of uremic toxins and development of severe kidney injury are demonstrated in 5/6- but not unilateral nephrectomized mice [8,9,22]. However, chronic exogenous addition of uremic toxins produces moderate renal fibrosis without changes in serum blood urea nitrogen (BUN) and creatinine in unilateral nephrectomized mice, implying an active role of uremic toxins directly or indirectly in pathophysiological changes [22]. To extend the scope of understanding with regard to CNS complications in CKD, a unilateral nephrectomized mice model was established in order to investigate the possible pathological role PCS plays in CNS through daily administration, as well as analyze any neurobehavioral changes and molecular mechanisms involved.

## 2. Results

### 2.1. PCS Caused Behavioral Changes

**Figure 1:**
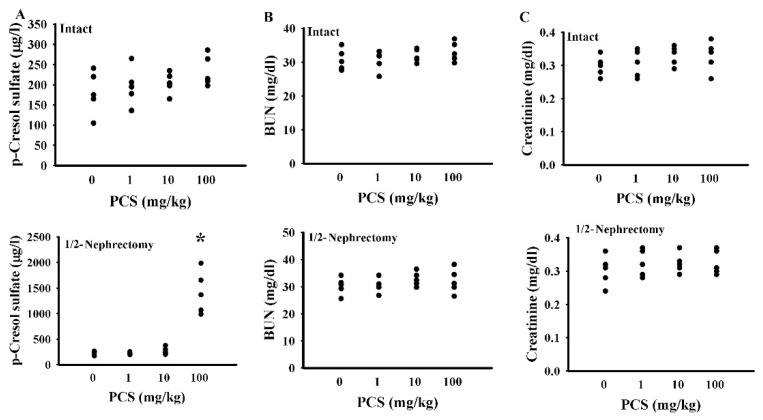
p-Cresol sulfate (PCS) administration caused PCS serum accumulation in nephrectomized mice. The intact and unilateral nephrectomized (1/2-Nephrectomy) mice were intraperitoneally injected with various doses of PCS for 7 weeks. The blood was collected and subjected to the measurement of PCS (**A**), blood urea nitrogen (BUN) (**B**), and creatinine (**C**). * *p* < 0.05 vs. control group without PCS, *n* = 5.

**Figure 2:**
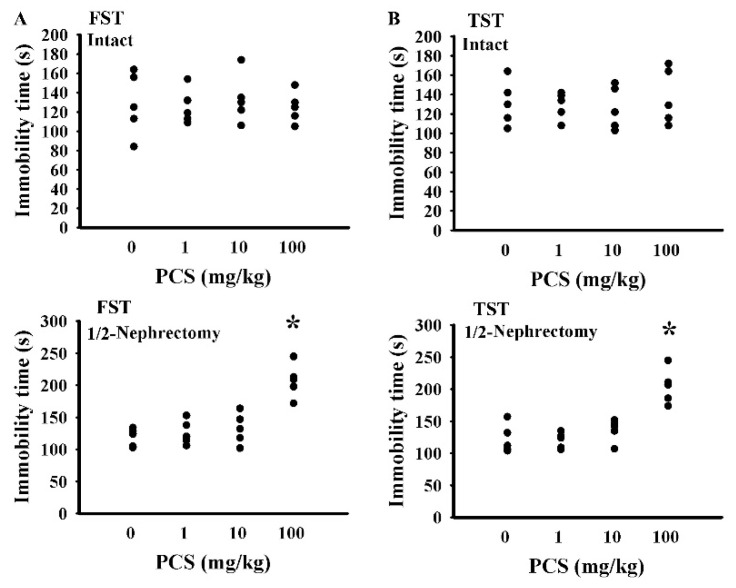
PCS caused alterations in nephrectomized mice. The intact and unilateral nephrectomized (1/2-Nephrectomy) mice were intraperitoneally injected with various doses of PCS for 7 weeks. The forced swimming test (FST) (**A**) and tail suspension test (TST) (**B**) were conducted and the duration of immobility was recorded. * *p* < 0.05 vs. control group without PCS, *n* = 5.

To investigate the potential effects of PCS on CNS changes, mice with a unilateral nephrectomy and the intact controls were intraperitoneally injected with various doses of PCS for 7 weeks. At a dose of 100 mg/kg/day, the PCS-exposed unilateral nephrectomized mice showed an elevated serum concentration of PCS (Figure 1A), while presenting normal ranges of serum BUN (Figure 1B) and creatinine (Figure 1C). Additionally, the mice showed an increased immobility time in the forced swimming test (FST) (Figure 2A) and tail suspension test (TST) (Figure 2B), implying abnormalities in neurobehaviors. Unexpectedly, the aforementioned alterations were not found in PCS-exposed, kidney-intact mice (Figure 1 and Figure 2). Therefore, subsequent studies were conducted in unilateral nephrectomized mice with PCS at a dose of 100 mg/kg.

**Figure 3:**
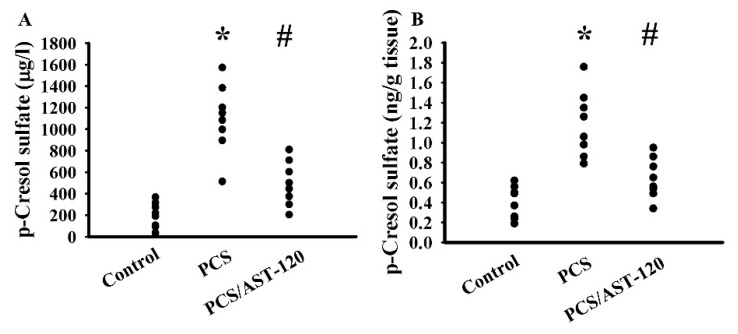
Peripheral PCS treatments increased its CNS distribution. Unilateral nephrectomized mice were intraperitoneally injected with PCS (0, Control; and 100 mg/kg, PCS) and the PCS-injected mice were orally given with AST-120 (400 mg/kg, PCS/AST-120) for 7 weeks. The blood (**A**) and prefrontal cortical tissues (**B**) were isolated and subjected to the measurement of PCS. * *p* < 0.05 vs. control group and # *p* < 0.05 vs. PCS group, *n* = 8.

**Figure 4:**
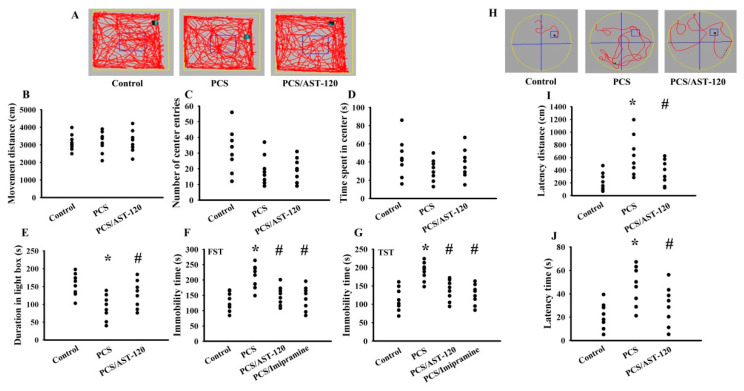
PCS caused behavioral alterations. Unilateral nephrectomized mice were intraperitoneally injected with PCS (0, Control; and 100 mg/kg, PCS) and the PCS-injected mice were orally given with AST-120 (400 mg/kg, PCS/AST-120) for 7 weeks. The tracking paths (**A**), distance in movement of spontaneous locomotor activity (**B**), numbers of central zone entries (**C**), and the time spent in the central zone (**D**) was evaluated by the open field test. The duration of light preference was evaluated by the light/dark box test (**E**). An FST was conducted and the duration of immobility was recorded (**F**). A TST was performed and the duration of immobility was recorded (**G**). Antidepressant imipramine (20 mg/kg) was intraperitoneally administrated 1 h prior to the FST (**F**) and TST (**G**). After training for consecutive 3 days, the tracking paths (**H**), distance (**I**), and time (**J**) required to reach the hidden platform were recorded for the Morris water maze test. * *p* < 0.05 vs. control group and # *p* < 0.05 vs. PCS group, *n* = 8.

During the course of the study, mice body mass and food intake were found not to be different amongst the three groups (data not shown). Treatment with PCS increased its presence in the serum (Figure 3A) and prefrontal cortical tissues (Figure 3B), while the increments were alleviated by uremic toxin absorbent AST-120 (Figure 3). PCS had a negligible effect on tracking paths (Figure 4A), movement distance (Figure 4B), numbers of central zone entries (Figure 4C), and time spent in the central zone (Figure 4D) in the open field test, but caused a reduction in the light box staying in the light/dark box test (Figure 4E), as well as an increase in immobility time in the FST (Figure 4F) and TST (Figure 4G). Furthermore, the clinical antidepressant imipramine decreased the PCS-increased immobility time in the FST (Figure 4F) and TST (Figure 4G). In the Morris water maze test, longer tracking paths (Figure 4H), escape latency distance (Figure 4I), and escape latency time (Figure 4J) were all identified in PCS mice. AST-120 alleviated those behavioral changes (Figure 4). Therefore, chronic exposure to PCS is believed to have an effect on the development of anxiety-like behavior, depression-like behavior, and spatial memory and learning impairment. Since the accumulated PCS can be detected in the prefrontal cortical tissue, which is the main target of anxiety-like behavior, depression-like behavior, and cognitive impairment [23], the prefrontal cortical tissues were used for subsequent biochemical analyses.

### 2.2. PCS Impaired Neuronal Survival and Neural Stem Cells

**Figure 5:**
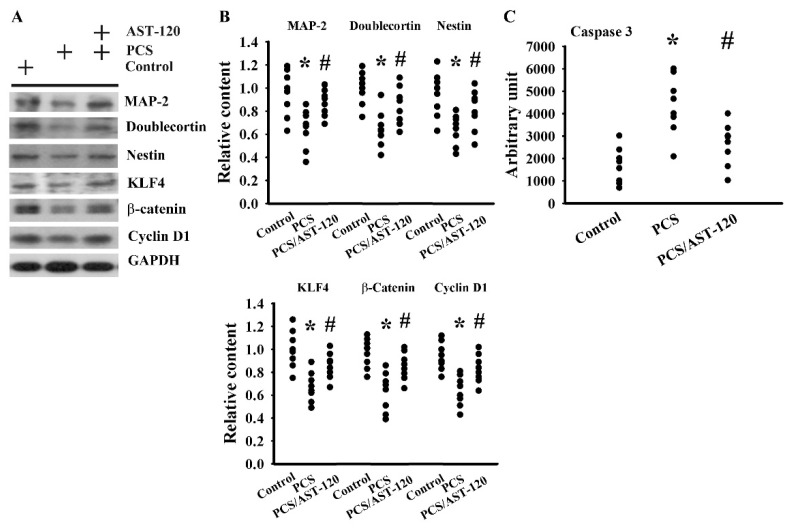
PCS decreased the parameters of neuronal cell survival and neural stem cells. Unilateral nephrectomized mice were intraperitoneally injected with PCS (0, Control; and 100 mg/kg, PCS) and the PCS-injected mice were orally given with AST-120 (400 mg/kg, PCS/AST-120) for 7 weeks. Proteins were extracted from the isolated prefrontal cortical tissues and subjected to a Western blot test with the indicated antibodies. Representative blots (**A**) and the quantitative data (**B**) are shown. Proteins were extracted from the isolated prefrontal cortical tissues and subjected to an enzymatic reaction for the measurement of caspase 3 activity (**C**). * *p* < 0.05 vs. control group and # *p* < 0.05 vs. PCS group, *n* = 8.

Neuronal degeneration, impaired neurogenesis, and cell death frequently occur in CNS diseases [24]. To elicit the effects of PCS on CNS, molecules related to neuronal cell survival, neural stem cells, and apoptosis were examined through Western blotting and an enzymatic assay. It was discovered that the protein content of neuronal cell-associated microtubule-associated protein 2 (MAP-2) decreased in PCS mice (Figure 5A,B). Parallel reductions were noted in the protein contents of the stem/progenitor biochemical markers of the neural lineage, including doublecortin, nestin, and KLF4, as well as cell proliferation-associated β-catenin and cyclin D1 (Figure 5A,B). On the contrary, caspase 3 activity was elevated (Figure 5C). AST-120 alleviated the protein reduction and caspase 3 activation in PCS mice (Figure 5). These findings indicate that PCS had a negative effects on neuronal cells and neural stem cells, as well as promoted cell apoptosis, while the alterations can be alleviated by AST-120.

### 2.3. PCS Impaired Neurotrophins and Neurotransmitters

**Figure 6:**
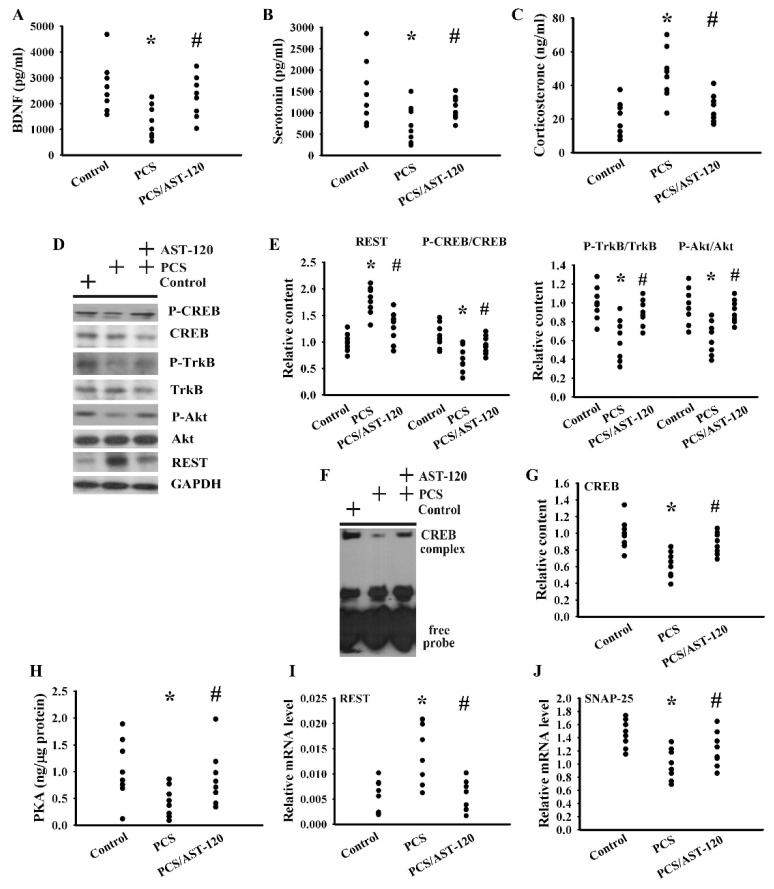
PCS decreased the parameters of the neurotrophins. Unilateral nephrectomized mice were intraperitoneally injected with PCS (0, Control; and 100 mg/kg, PCS) and the PCS-injected mice were orally given with AST-120 (400 mg/kg, PCS/AST-120) for 7 weeks. The serum samples were collected and subjected to the measurement of the BDNF (**A**), serotonin (**B**), and corticosterone (**C**) levels. Proteins were extracted from the isolated prefrontal cortical tissues and subjected to a Western blot test with the indicated antibodies. Representative blots (**D**) and the quantitative data (**E**) are shown. Nuclear proteins were extracted from the isolated prefrontal cortical tissues and subjected to EMSA for the measurement of CREB DNA-binding activity. Representative blots (**F**) and the quantitative data (**G**) are shown. (**H**) The prefrontal cortical tissues were isolated and subjected to the measurement of PKA activity. Total RNAs were extracted from the isolated prefrontal cortical tissues and subjected to quantitative RT-PCR for the measurement of the REST (**I**) and SNAP-25 (**J**) mRNA level. * *p* < 0.05 vs. control group and # *p* < 0.05 vs. PCS group, *n* = 8.

The impairment of brain-derived neurotrophic factor (BDNF) signaling and serotonergic neurotransmission, as well as the activation of corticosterone action, have been implicated in the pathogenesis of CNS diseases, particularly depression [25,26,27]. Serum levels of BDNF (Figure 6A) and serotonin (Figure 6B) were decreased, while that of corticosterone (Figure 6C) was increased in PCS mice. PCS decreased the protein phosphorylation of the BDNF receptor tropomyosin-related kinase receptor type B (TrkB) and transcription factor cAMP response element-binding protein (CREB) (Figure 6D,E), as well as the DNA binding activity of CREB (Figure 6F,G). Moreover, PCS also had an inhibitory effect on Akt phosphorylation (Figure 6D,E) and PKA activity (Figure 6H), which are crucial upstream activators of CREB. A transcriptional repressor, the repressor element-1 silencing transcription factor (REST), controls the gene expression of neurotrophins and neurotransmitters and has been implicated in mood disorders [28]. PCS had a promoting effect on the expression of REST protein (Figure 6D,E) and mRNA (Figure 6I), concurrent with the reduction in the mRNA level of the REST downstream synaptosomal-associated protein 25 (SNAP-25) gene (Figure 6J). The alterations caused by PCS were alleviated by AST-120 (Figure 6). These findings suggest that PCS is shown to have inhibitory effects on both BDNF and serotonin expression, while inducing an effect on corticosterone release, and that intracellular signaling and the reduction in BDNF and serotonin expression can be improved by AST-120.

### 2.4. PCS Induced Oxidative Stress

**Figure 7:**
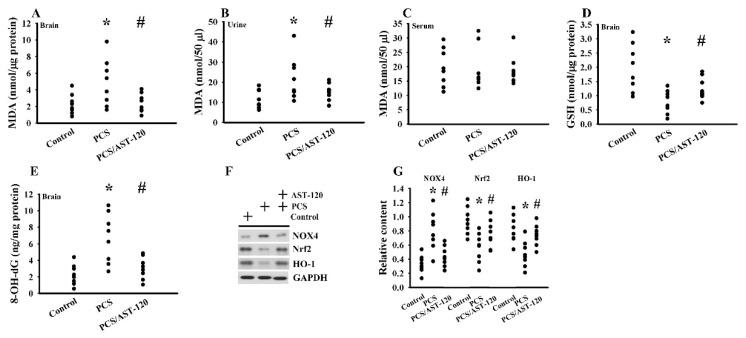
PCS induced oxidative stress. Unilateral nephrectomized mice were intraperitoneally injected with PCS (0, Control; and 100 mg/kg, PCS) and the PCS-injected mice were orally given with AST-120 (400 mg/kg, PCS/AST-120) for 7 weeks. The prefrontal cortical tissues (**A**), urine (**B**, 24 h), and blood (**C**) were collected and subjected to the measurement of MDA levels. The prefrontal cortical tissues were collected and subjected to the measurement of GSH content (**D**). The prefrontal cortical tissues were collected and subjected to the measurement of 8-OH-dG content (**E**). Proteins were extracted from the isolated prefrontal cortical tissues and subjected to a Western blot test with the indicated antibodies. Representative blots (**F**) and the quantitative data (**G**) are shown. * *p* < 0.05 vs. control group and # *p* < 0.05 vs. PCS group, *n* = 8.

**Figure 8:**
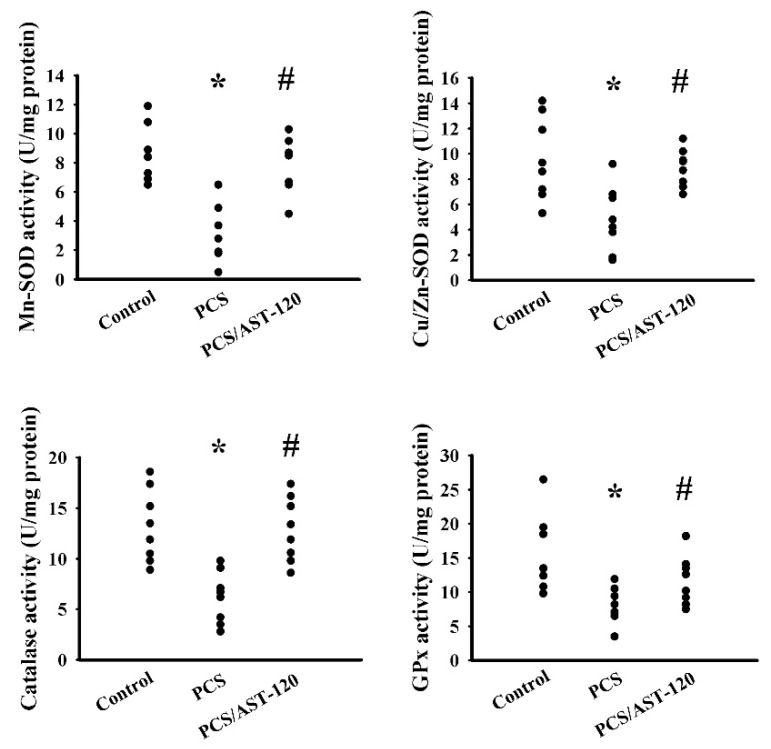
PCS decreased antioxidant enzyme activities. Unilateral nephrectomized mice were intraperitoneally injected with PCS (0, Control; and 100 mg/kg, PCS) and the PCS-injected mice were orally given with AST-120 (400 mg/kg, PCS/AST-120) for 7 weeks. The prefrontal cortical tissues were collected and subjected to the measurement of Mn-SOD activity, Cu/Zn-SOD activity, catalase activity, and GPx activity. * *p* < 0.05 vs. control group and # *p* < 0.05 vs. PCS group, *n* = 8.

Oxidative stress has an impact on neural function and the overwhelmed oxidative stress contributes to the pathogenesis of CNS diseases [29,30,31,32,33]. An elevated level of malondialdehyde (MDA) was noted in the prefrontal cortical tissues (Figure 7A) and urine (Figure 7B), but not serum (Figure 7C), in PCS mice. Besides, PCS exposure decreased the glutathione (GSH) content (Figure 7D), increased the 8-hydroxy-2-deoxyguanosine (8-OH-dG) content (Figure 7E) and NADPH oxidase-4 (NOX4) (Figure 7F,G), and lowered the NF-E2-related factor (Nrf2), heme oxygenase-1 (HO-1) (Figure 7F,G), manganese-superoxidase dismutase (Mn-SOD) activity, copper/zinc-superoxidase dismutase (Cu/Zn-SOD) activity, catalase activity, and glutathione peroxidase (GPx) activity (Figure 8) in the prefrontal cortical tissues. The changes could be reduced by AST-120 (Figure 7 and Figure 8). These data suggest a pro-oxidative effect for PCS, and a reversal effect for AST-120.

### 2.5. PCS Induced Neuroinflammation

**Figure 9:**
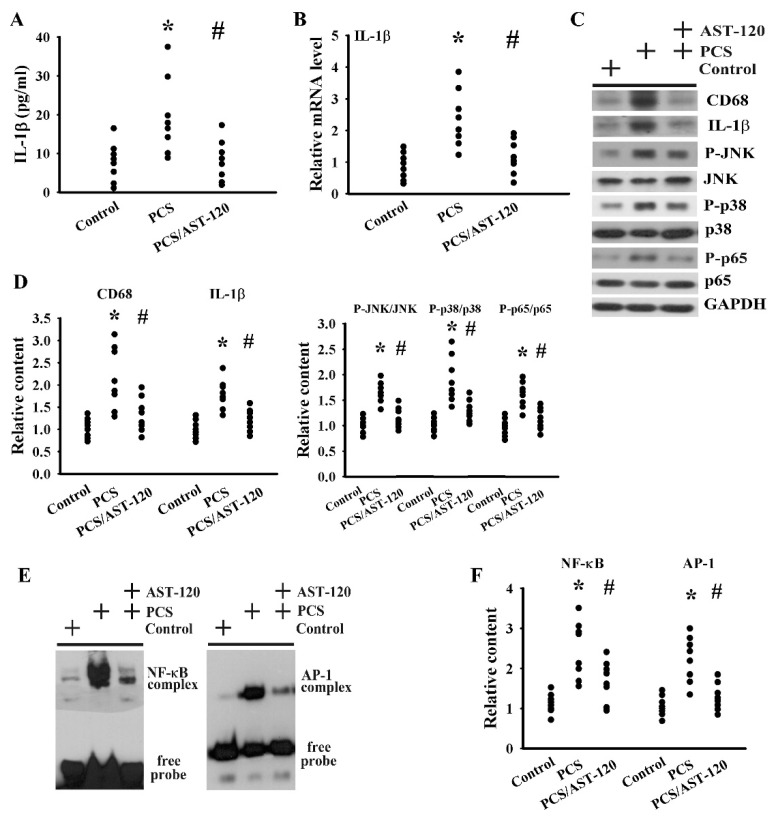
PCS induced neuroinflammation. Unilateral nephrectomized mice were intraperitoneally injected with PCS (0, Control; and 100 mg/kg, PCS) and the PCS-injected mice were orally given with AST-120 (400 mg/kg, PCS/AST-120) for 7 weeks. (**A**) The serum samples were collected and subjected to the measurement of IL-1β levels. (**B**) Total RNAs were extracted from the isolated prefrontal cortical tissues and subjected to quantitative RT-PCR for the measurement of IL-1β mRNA levels. Proteins were extracted from the isolated prefrontal cortical tissues and subjected to a Western blot test with the indicated antibodies. Representative blots (**C**) and the quantitative data (**D**) are shown. Nuclear proteins were extracted from the isolated prefrontal cortical tissues and subjected to EMSA for the measurement of NF-κB and AP-1 DNA-binding activity. Representative blots (**E**) and the quantitative data (**F**) are shown. * *p* < 0.05 vs. control group and # *p* < 0.05 vs. PCS group, *n* = 8.

Neuroinflammation represents an active player in the pathogenesis of CNS diseases; therefore, anti-inflammatory therapy is of benefit for disease control [23,31,32,33]. Serum levels of IL-1β protein (Figure 9A) and prefrontal cortical tissue levels of IL-1β mRNA (Figure 9B) and IL-1β protein (Figure 9C,D) were elevated in PCS mice. The actions of PCS on the prefrontal cortical tissues were accompanied by an increased protein expression of monocyte/macrophage/microglia-associated CD68, protein phosphorylation of p38, c-Jun N-terminal Kinase (JNK), and p65 (Figure 9C,D), along with the DNA-binding activity of NF-κB and AP-1 (Figure 9E,F), crucial signaling molecules, and transcription factors in neuroinflammation. The alterations of the neuroinflammation-associated molecules were alleviated by AST-120 (Figure 9). These data suggest that PCS was able to induce neuroinflammation.

## 3. Discussion

CKD has been well associated with several CNS diseases [2,3,4]. Despite the clinical and experimental studies suggesting their association, the underlying causes of their association remain unclear, but may be related to the accumulation of uremic toxins. In this study, we hypothesized that it might arise from the accumulation of uremic toxins, which consequently caused neurological complications in CKD. In unilateral nephrectomized mice, we provided evidence showing that the protein-bound uremic toxin PCS is one such surrogate of the association. Serum PCS concentration was progressively increased in unilateral nephrectomized mice after PCS administration at a dose of 100 mg/kg/day. Further, there was an apparent deposition of PCS in the prefrontal cortical tissues associated with several abnormal behaviors, such as depression, anxiety, and cognitive impairment. However, PCS accumulation and behavioral changes were not observed at lower doses at 1 and 10 mg/kg/day in unilateral nephrectomy nor at doses at 1, 10, and 100 mg/kg/day in intact kidneys. These changes were alleviated by uremic toxin absorbent AST-120. The current findings extended our understanding of CNS complications, such as depression, anxiety, and cognitive impairment, in patients with CKD, and highlight a pathogenic role of the uremic toxin PCS in the development of CNS diseases in CKD.

In rodent studies, the Y maze, elevated plus maze, light/dark box, open field test, Morris water maze test, object recognition test, sucrose preference test, FST, and TST are all commonly used for the evaluation of neurobehaviors with underlying CNS diseases. Amongst them, the light/dark box test is used for measuring anxiety. The open field test is widely used for the evaluation of rodent exploration activity, locomotor activity, and anxiety. The Morris water maze test is commonly used in behavioral assays for memory and learning. In addition, depression behavior is determined by the FST and TST [24,29,34,35,36]. Herein, data regarding behavioral evaluation have suggested an induction of depression-like and anxiety-like behavior, as well as spatial memory and learning impairment in PCS mice. Our findings run parallel with the clinical observation symptoms of depression, anxiety, and cognitive impairment, which are implicated in patients with CKD [2,3]. That is to say that PCS is a causative molecule for the development of CNS complications in CKD patients. However, its generalized abnormality in several types of behavioral evaluations weakened the association specificity of PCS and neurological complications in unilateral nephrectomized mice. That might arise from its general neurotoxic actions, exposure doses, exposure times, or unidentified confounding factors. Although there were limitations, the current findings still highlighted a potential contribution of PCS in the development of depression in this rodent model. The etiology of depression is multifactorial, including genetic, environmental, and dietary factors, particularly chronic stress and diseases [5,6,26,28]. Accumulating evidence highlights the pathogenic roles of oxidative stress, neuroinflammation, impaired neurogenesis, as well as dysregulated neurotransmitters and neurohormonal pathways in the development and progression of depression [6,23,24,25,29]. Clinically, patients with depression are routinely prescribed with serotonin-norepinephrine reuptake inhibitors, selective serotonin reuptake inhibitors, monoamine oxidase inhibitors, or tricyclic antidepressants for symptom relief [37]. Apart from data from behavioral, biochemical, and molecular studies, PCS-induced depression-like behavior was further confirmed by the symptom relief after the intervention of the clinical antidepressant imipramine. It should be noted that before its translational implication, additional study designs and experiments are required.

The molecular and cellular bases of depression, oxidative stress, and neuroinflammation have a central role to initiate a series of biochemical events, which result in a negative impact on neural function and, consequently, behavioral change [23,29,30,31,32,33]. The resultant neuronal cell apoptosis and autophagy, dysregulated neurochemicals, impaired neurogenesis, disturbed synaptic connection, and disrupted blood–brain barrier all play substantial roles, while contributing to depression [23,26,29,30]. In the present study, PCS exhibited biological potential in inducing oxidative stress and neuroinflammation in nephrectomized mice, as evidenced by decreased brain antioxidant enzyme activities and Nrf2/HO-1, increased NOX4, elevated 8-OH-dG and MDA, as well as brain and serum levels of proinflammatory cytokine IL-1β. Because the elevation of NOX4 has been implicated in neurological disorder-associated free radical generation [38], our findings suggested that the elevation of NOX4 and reduction of Nrf2/HO-1 are causes of PCS-induced oxidative stress. Data from the Western blot test and enzymatic assay demonstrated a reduction in neuronal cell survival, cell proliferative potential, and neurogenesis, as well as an increase in cell apoptosis caused by PCS. There was also an inhibition of BDNF and serotonin neurotrophic signaling, and activation of the corticosterone damaging action. It appeared that PCS caused oxidative stress and neuroinflammation as well as changes in neurotrophins, neurotransmitters, and stress hormones. Additionally, the increased expression of the transcriptional repressor REST represented alternative mechanisms of PCS to silence the gene expression of both the neurotrophins and neurotransmitters. Despite the crosstalk between these biochemical events being complicated, current findings indicate that PCS may initiate, integrate, and establish a pathogenic environment predisposing the onset of CNS diseases, particularly depression, in rodents with unilateral nephrectomy.

In the periphery, PCS has effects on oxidative stress, inflammation, mitochondrial activity, vascular function, and cell viability [15,16,17,18,19,20,21]. In the prefrontal cortical tissues we found that PCS increased the expression and activities of redox-sensitive and proinflammatory molecules, such as IL-1β, JNK, p38, p65, NF-κB, and AP-1, along with a reduction in circulating BDNF and serotonin as well as an increase in corticosterone. Microglia are the central players regarding neuroinflammation in the CNS [39]. Although the specific and corresponding immunocompetent cells were not identified in detail, the elevation of microglia-associated CD68 protein implied a potential role that microglia plays in PCS-induced neuroinflammation. CNS glial cells are targets of uremic toxins for free radical generation and proinflammatory cytokine expression [40]. Besides, peripheral corticosterone administration is an alternative approach for the induction of depression-like behavior in the rodents [27]. Whether the CNS effects of PCS came from its CNS accumulation, or secondarily due to its peripheral actions, was not clear, so further investigation is necessary. Another interesting finding of the current study was the lack of detectable changes in Aβ and Tau phosphorylation (data not shown), although data of the Morris water maze test revealed an impairment of cognition. Therefore, the current findings remind us of crucial issues that should be investigated in the near future. What happens to the striatum, hippocampus, and midbrain after PCS exposure because they are involved in various behaviors? What are the neurological consequences between acute and chronic exposure that may be caused by PCS or cessation of PCS exposure? We thought that PCS could cause apparent signs of additional chronic neurodegenerative diseases in case of distinct exposure programs. Further studies are necessary to strengthen the current findings and extend biological implications of PCS in CKD-associated neurological complications.

As with PCS, indoxyl sulfate is another well-known protein-bound uremic toxin associated with worsening outcomes in CKD patients. Accordingly, the pathogenic role of indoxyl sulfate in CKD-associated CNS complications is also highly expected. Indeed, our concurrent studies also observed the prooxidant, inflammatory, and neurodegenerative actions of indoxyl sulfate with similar and distinct molecular and cellular profiles of PCS (under preparation). Indoxyl sulfate and PCS are the bioactive metabolites of gut microbiota, synthesized through the metabolism of amino acids. When the ingested protein is decomposed, it will produce tryptophan and tyrosine, respectively. The function of gut bacteria is to metabolize it into indole and p-cresol. These two substances, after being absorbed by the intestinal cells, enter the blood stream and travel to the liver cells, where they are metabolized to indoxyl sulfate and PCS [41,42]. In contrast, *Clostridium sporogenes* could metabolize tryptophan into indole and subsequently 3-indolepropionic acid, a highly potent neuroprotective antioxidant that scavenges hydroxyl radicals [42,43]. CKD patients are frequently associated with gut dysbiosis and the elevation of circulating indoxyl sulfate and PCS [14,44]. Probiotics or nutritional therapy are of benefit for improving gut dysbiosis and decreasing circulating uremic toxins, oxidative stress, and inflammation [6,45]. Since indoxyl sulfate and PCS remain difficult to remove by hemodialysis, gut microbiota could be an alternative target for reducing the circulating indoxyl sulfate, PCS levels, and their toxicity in CKD patients, with the aim of slowing down the progression of the disease and decreasing any CNS complications.

Symptoms of depression, anxiety, and cognitive impairment are linked with CKD patients who are undergoing maintenance hemodialysis, and the levels of uremic toxins, such as PCS and indoxyl sulfate, exhibit a strong association with disease progression [2,46]. Through this study, we have provided experimental evidence showing the pathogenic effect of PCS for CNS diseases in nephrectomized mice. Depression-like and anxiety-like behavior, as well as cognitive impairment caused by PCS, were accompanied by PCS CNS accumulation, impaired neuronal cell survival, and neurogenesis, apoptosis, disturbed BDNF, serotonin, corticosterone, REST expression, oxidative stress, and neuroinflammation in nephrectomized mice. Although there remained several limitations in our experiments, the protein-bound uremic toxin PCS is a proposed pathogenic target for the link between CKD and CNS diseases in CKD, while the chelation of PCS or reduction of PCS synthesis by probiotics or nutritional therapy may represent therapeutic strategies for CKD-associated CNS complications.

## 4. Materials and Methods

### 4.1. Study Animals

The Animal Experimental Committee of Taichung Veterans General Hospital reviewed and approved the protocols of this animal study (IACUC approval code: La-1051370, IACUC approval date: 8 February 2016). Ten-week-old male C57BL/6 mice (88 mice in total) were housed in a controlled animal facility. Sixty-eight mice were subjected to unilateral nephrectomy under isoflurane anesthesia 1 week prior to treatments [22]. The remaining twenty mice were the kidney-intact controls. For the dose effect study (Figure 1 and Figure 2), the intact (*n* = 20) and unilateral nephrectomized (*n* = 20) mice were divided into four groups (*n* = 5 per group) receiving various doses of PCS (0, 1, 10, and 100 mg/kg/day) intraperitoneally. The mice were used for all indicated assays. For intervention (Figure 3, Figure 4, Figure 5, Figure 6, Figure 7, Figure 8 and Figure 9), thirty-two unilateral nephrectomized mice were treated daily with PCS (100 mg/kg) via an intraperitoneal injection, while sixteen unilateral nephrectomized mice received an administration of normal saline. Half of the PCS mice (*n* = 16) were concurrently given spherical carbonaceous absorbent AST-120 (400 mg/kg) via oral gavage. According to the pilot findings, based on an FST and TST evaluation, the PCS mice showed behavioral change starting from 5 weeks after administration (data not shown). Therefore, the study course was scheduled to 7 weeks for treatments. To evaluate the behavioral changes, mice in the intervention study were randomly allocated into two groups. Half of the mice were allocated for the evaluation of the open field test and Morris water maze test, while the rest half of mice were allocated for the evaluation of the TST, FST, and light/dark box test (*n* = 8 per group). At the end of the behavioral evaluation (7 weeks after treatment), the mice were euthanized and the prefrontal cortical tissues, blood, and urine (24 h) were collected for further analyses.

### 4.2. Measurement of PCS

The blood and prefrontal cortical tissues were isolated and homogenized with normal saline. Two hundred microliters of the homogenates were mixed with acetonitrile (400 μL), vortexed, and centrifuged at 10,000 rpm for 10 min. The supernatants were then diluted with distilled water and subjected to the measurement of PCS using high-performance liquid chromatography [13].

### 4.3. Behavioral Evaluation

The behavioral evaluation was conducted according to our previously reported methods by technicians who were blind to the treatments [24,29,34,36]. In the open field test, mice were placed on an apparatus 30 cm in length and 30 cm in width. The travel distance for a period of 30 min was recorded by a video camera. For the measurement of the FST, mice were forced to swim for 5 min, with their immobility time then recorded. The TST was conducted by suspending the mice by its tail for 6 min and recording their immobility time. To evaluate the effect of the tricyclic antidepressant imipramine, a dose of 20 mg/kg was intraperitoneally administrated 1 h prior to the FST and TST evaluations. A half-light and half-dark apparatus 40 cm in length, 30 cm in width, and 30 cm in height was used in the light/dark box test. The time of mice entry and exit from the light box during a 5 min period was recorded. A platform (10 cm in diameter) was placed 1 cm below the water and 50 cm away from the wall of a water maze, which was 150 cm in diameter and 60 cm in depth. Mice were trained to find the hidden platform for three consecutive days. Afterwards, the Morris water maze test was conducted by recording the time and distance for each of the mice to reach the hidden platform.

### 4.4. Western Blot

The separation of proteins obtained from the prefrontal cortical tissues was performed by a standard SDS-PAGE. The interested proteins were identified by corresponding antibodies, and visualized with enhanced chemiluminescence, prior to being quantified by a densitometer. The targets of recognizing antibodies were MAP-2 (sc-74421), nestin (sc-23927), glyceraldehyde-3-phosphate dehydrogenase (GAPDH, sc-47724), cyclin D1 (sc-8396), doublecortin (sc-271390), KLF4 (sc-166238), TrkB (sc-8316), CD68 (sc-20060), Akt (sc-8312), phosphorylated Akt (sc-271966), p38 (sc-7972), phosphorylated p38 (sc-17852-R), JNK (sc-7345), phosphorylated JNK (sc-6254), p65 (sc-372), phosphorylated p65 (sc-136548), β-catenin (sc-7963), REST (sc-374661) (Santa Cruz Biotechnology, Santa Cruz, CA, USA), phosphorylated TrkB (ab131483), NOX4 (ab109225), Nrf2 (ab137550), HO-1 (ab13248) (Abcam, Cambridge, UK), CREB (#9104), and phosphorylated CREB (#9191) (Cell Signaling, Danvers, MA, USA).

### 4.5. RNA Isolation and Quantitative Real-Time Reverse Transcriptase Polymerase Chain Reaction (RT-PCR)

The total RNAs were extracted from the resected prefrontal cortical tissues and subjected to cDNA synthesis according to our previously reported methods [34]. The ABI StepOne^TM^ machine (Applied Biosystems, Foster City, CA, USA) was used to amplify and measure the level of mRNA expression, with the levels of specific mRNA being calculated by the ΔΔCT method and normalized with β-actin. Oligonucleotide sequences used in the PCR were 5′-TCAGGCAGGCAGTATCACTC and 5′-AGCTCATATGGGTCCGACAG for IL-1β; 5′-ACATGCGTAATGAACTGGAG and 5′-GAGCAAGGCGAACAACTGGAACG for SNAP-25; 5′-CCTGCAGCAAGTGCAACTAC and 5′-CTTCTGAGAGCTTGAGTAAGG for REST; 5′-CACGATGGAGGGGCCGGACTCATC and 5′-TAAAGACCTCTATGCCAACACAGT for β-actin.

### 4.6. Measurement of BDNF, Serotonin, IL-1β, Corticosterone, and Serum Biochemical Content

Equal amounts of serum samples were allocated to 96-well plates for the measurement of BDNF, serotonin, IL-1β, and corticosterone using the corresponding Enzyme-Linked Immunosorbent Assay (ELISA) kits according to the manufacturer’s instructions (R&D Systems, Minneapolis, MN, USA). Serum levels of BUN and creatinine were measured by automated standardized procedures (Roche Hitachi, Mannheim, Germany).

### 4.7. Measurement of Protein Kinase A (PKA) Activity

Proteins were extracted from the resected prefrontal cortical tissues and subjected to PKA activity measurement using a commercially available PKA kinase activity assay kit (Enzo Life Sciences, NY, USA). The calculated activity was expressed as the amount of active PKA protein (ng/μg protein).

### 4.8. Preparation of Nuclear Extracts and Electrophoretic Mobility Shift Assay (EMSA)

A commercial assay kit of nuclear protein extraction (NE-PER Nuclear and Cytoplasmic Extraction Kit, ThermoFisher Scientific, IL, USA) was used to extract nuclear proteins from the resected prefrontal cortical tissues. The measurement of DNA-binding activity for NF-κB, AP-1, and CREB was conducted by an EMSA assay kit (LightShift^TM^ Chemiluminescent EMSA Kit, ThermoFisher Scientific, IL, USA). The reactive DNA/protein complexes were detected using chemiluminescence reagents. The oligonucleotides recognizing NF-κB, AP-1, and CREB were 5′-AGTTGAGGGGACTTTCCCAGGC, 5′-CGCTTGATGAGTCAGCCGGAA, and 5′-AGAGATTGCCTGACGTCAGAGAGCTAG, respectively.

### 4.9. Measurement of Lipid Peroxidation

The prefrontal cortical tissues, serum, and 24 h urine samples were collected and isolated. The levels of lipid peroxidation products were measured using a Thiobarbituric Acid Reactive Substances (TBARS) assay kit (ZeptoMetrix, Buffalo, NY, USA). The calculated levels of the lipid peroxidation products were expressed as MDA equivalents according to the manufacturer’s instructions.

### 4.10. Measurement of Antioxidant Enzyme Activity

The prefrontal cortical tissues were collected. The levels of reduced GSH and activities of Mn-SOD, Cu/Zn-SOD, catalase, and GPx were measured using commercially available assay kits (Cayman, Ann Arbor, MI, USA).

### 4.11. Measurement of 8-OH-dG

The prefrontal cortical tissues were collected and isolated. The levels of 8-OH-dG were measured using commercially available assay kits (8-hydroxy 2 deoxyguanosine ELISA Kit, Cayman, Ann Arbor, MI, USA).

### 4.12. Caspase 3 Activity Assay

The prefrontal cortical tissues were collected and isolated. Tissues were homogenized and the proteins were subjected to enzymatic reaction of caspase 3 using the Caspase Fluorometric Assay kit (BioVision, Mountain View, CA, USA). The liberated fluorescent AMC moiety was measured with a fluorometer (E_x_ 380 nm and E_m_ 460 nm) and the fluorescence signals were normalized by protein contents.

### 4.13. Statistical Analysis

A one-way analysis of variance (ANOVA) was performed to compare effects between groups. Individual differences were determined using a Bonferroni or Tukey post-hoc test. A level of *p* < 0.05 was considered statistically significant.

## Abbreviations

BDNF	Brain-Derived Neurotrophic Factor
CREB	cAMP Response Element-Binding Protein
CNS	Central Nervous System
CKD	Chronic Kidney Disease
JNK	c-Jun N-terminal Kinase
EMSA	Electrophoretic Mobility Shift Assay
ELISA	Enzyme-Linked Immunosorbent Assay
FST	Forced Swimming Test
GAPDH	Glyceraldehyde-3-Phosphate Dehydrogenase
MDA	Malondialdehyde
MAP-2	Microtubule-Associated Protein 2
PCS	p-Cresol Sulfate
PKA	Protein Kinase A
REST	Repressor Element-1 Silencing Transcription Factor
SNAP-25	Synaptosomal-Associated Protein 25
TST	Tail Suspension Test
TBARS	Thiobarbituric Acid Reactive Substances
TrkB	Tropomyosin-Related Kinase Receptor Type B
